# What Are the Most Distressing Aspects of Experiencing Elder Abuse? Findings From a Qualitative Study With Victims

**DOI:** 10.1093/geroni/igaa057.1050

**Published:** 2020-12-16

**Authors:** Jessica Hsieh, David Burnes, Clara Scher, Paula Zanotti, Chelsie Burchett, Jo Anne Sirey, Mark Lachs

**Affiliations:** 1 University of Toronto, Toronto, Ontario, Canada; 2 Weill Cornell Medicine, White Plains, New York, United States; 3 Weill Cornell Medicine, New York, New York, United States; 4 Weill Cornell Medicine, New York City, New York, United States

## Abstract

Adult protective services and other community-based agencies respond to hundreds of thousands of elder abuse cases annually in the United States; however, few studies include elder abuse victims’ voices. This study explored the most distressing aspects of elder abuse, as identified by victims themselves; to date, this is the first known study on this topic. Guided by a phenomenological qualitative methodology, this study conducted in-person, semi-structured interviews with a sample of elder abuse victims (n = 30) recruited from a community-based elder abuse social service program in New York City. To enhance trustworthiness, two researchers independently analyzed transcript data to identify key transcript codes/themes. Distressing aspects of elder abuse were identified across three key domains, related to feelings of loss (50% of codes), threats/negative consequences (55%), and client-needs/system incongruity (14%). Specifically, the first theme represented outcomes related to loss of relationships (19% of ‘loss’ codes), personhood (16%), credibility (19%), faith/trust in others (38%), and finances (8%). The second theme looked at threats to physical self (34% of ‘threat’ codes), psyche (39%), and others, including the perpetrator (27%). The third theme focused on mismatches in client/system goals (50% of ‘incongruity’ codes) and legal system involvement (50%). The findings in this study provide a comprehensive and conceptually organized range of aspects to serve as infrastructure for the development of meaningful interventions to address the needs of victims. This study represents one of the largest efforts to understand and integrate the perspectives and needs of victims into elder abuse intervention practice/research to date.

